# The Selective Phosphodiesterase 4 Inhibitor Roflumilast and Phosphodiesterase 3/4 Inhibitor Pumafentrine Reduce Clinical Score and TNF Expression in Experimental Colitis in Mice

**DOI:** 10.1371/journal.pone.0056867

**Published:** 2013-02-28

**Authors:** Florian Rieder, Britta Siegmund, Daniela S. Bundschuh, Hans-Anton Lehr, Stefan Endres, Andreas Eigler

**Affiliations:** 1 Division of Clinical Pharmacology and Section of Gastroenterology, University of Munich, Munich, Germany; 2 Department of Internal Medicine I, University of Regensburg, Regensburg, Germany; 3 Medical Department I, Charité Universitätsmedizin, Campus Benjamin Franklin, Berlin, Germany; 4 Takeda Pharmaceuticals International GmbH, Zurich, Switzerland; 5 Institut Universitaire de Pathologie, Centre Hospitalier Universitaire Vaudois, Lausanne, Switzerland; Charité-University Medicine Berlin, Germany

## Abstract

**Objective:**

The specific inhibition of phosphodiesterase (PDE)4 and dual inhibition of PDE3 and PDE4 has been shown to decrease inflammation by suppression of pro-inflammatory cytokine synthesis. We examined the effect of roflumilast, a selective PDE4 inhibitor marketed for severe COPD, and the investigational compound pumafentrine, a dual PDE3/PDE4 inhibitor, in the preventive dextran sodium sulfate (DSS)-induced colitis model.

**Methods:**

The clinical score, colon length, histologic score and colon cytokine production from mice with DSS-induced colitis (3.5% DSS in drinking water for 11 days) receiving either roflumilast (1 or 5 mg/kg body weight/d p.o.) or pumafentrine (1.5 or 5 mg/kg/d p.o.) were determined and compared to vehicle treated control mice. In the pumafentrine-treated animals, splenocytes were analyzed for interferon-γ (IFNγ) production and CD69 expression.

**Results:**

Roflumilast treatment resulted in dose-dependent improvements of clinical score (weight loss, stool consistency and bleeding), colon length, and local tumor necrosis factor-α (TNFα) production in the colonic tissue. These findings, however, were not associated with an improvement of the histologic score. Administration of pumafentrine at 5 mg/kg/d alleviated the clinical score, the colon length shortening, and local TNFα production. *In vitro* stimulated splenocytes after *in vivo* treatment with pumafentrine showed a significantly lower state of activation and production of IFNγ compared to no treatment *in vivo*.

**Conclusions:**

These series of experiments document the ameliorating effect of roflumilast and pumafentrine on the clinical score and TNF expression of experimental colitis in mice.

## Introduction

Inflammatory bowel disease (IBD) is characterized by a disturbed balance of pro- and anti-inflammatory cytokines. Tumor necrosis factor-α (TNFα), which is elevated in the intestinal mucosa of both of its entities – Crohn’s disease (CD) and ulcerative colitis (UC) [1] plays a crucial role in the pathogenesis of IBD [2], a fact further underlined by the efficacious treatment of patients with CD and UC with anti-TNFα antibodies [3], [4], [5]. However, repeated administration of infliximab results in the production of auto-antibodies and antibodies against double-stranded DNA [3], [4]. In addition, patients treated with infliximab are at an increased risk of concomitant infectious complications secondary to the sustained immune suppression [6].

Among the agents known to inhibit pro-inflammatory cytokine production rather than to block its biological function are cyclic adenosine-3′,5′-monophosphate (cAMP)-elevating PDE inhibitors. A major PDE isoenzyme family in mononuclear inflammatory cells, the main source of TNFα production, is PDE4 [7]. The specific inhibition of PDE4 with rolipram is 500-fold more potent in suppressing TNFα synthesis in human mononuclear cells compared to pentoxifylline [8]. PDE4 inhibitors have shown efficacy in the treatment of several chronic inflammatory disorders, including experimental colitis [9], [10], [11]. Unfortunately, the clinical use of rolipram and other investigational PDE4 inhibitors was limited by its adverse effects profile [7], [12]. Another major PDE isoenzyme found in several human immune and inflammatory cells (T-cells, macrophages, dendritic cells) is PDE3 [13]. Interestingly, dual selective inhibition of PDE3 and PDE4 frequently leads to an over additive modulation of inflammatory cell functions compared to inhibition of either isoform alone [14], [15].

Roflumilast is an oral, once daily, PDE4 inhibitor marketed in the European Union and United States and several other countries for the treatment of severe COPD [15], [16], [17], [18]. *In vitro*, *in vivo* and clinical studies demonstrated an anti-inflammatory potential of roflumilast accompanied by a favorable tolerability profile compared to earlier PDE4 inhibitors [15], [16], [19], [20]. These anti-inflammatory effects of the PDE4 inhibitor may translate into the improved lung function and reduced rate of exacerbations in patients with moderate to severe COPD as recently documented in large-scale clinical trials. Roflumilast was generally well tolerated [17], [18], [21].

In the present study we investigated the effect of the PDE4 inhibitor roflumilast and the PDE3/4 inhibitor pumafentrine [22] in the preventive model of murine dextran sulphate sodium (DSS)-induced colitis [23]. DSS-induced colitis is the most frequently used model for IBD and is responsive to and predictive of drugs used for the treatment of IBD.

## Materials and Methods

### Mice

Female, 8-week old BALB/c mice (Harlan Winkelmann, Borchen, Germany) weighing 20 to 22 g were used in this study. The animals were housed in rooms at a controlled temperature and with light/dark (12h/12h) cycles. They were fed standard mice chow pellets and had access to tap water supplied in bottles. Mice were euthanized by cervical dislocation under isoflurane anesthesia (Forene; Abbott GmbH, Wiesbaden, Germany). Animal handling, clinical as well as histologic scoring of colitis were performed as treatment-blinded assessments. All experiments were approved by the regional animal study committee of the Ludwig-Maximilians University of Munich and were in agreement with the guidelines for the proper use of animals in biomedical research. All efforts were made to minimize suffering.

### Reagents

Roflumilast (3-cyclopropylmethoxy-4-difluoromethoxy-N-^hello^3,5-di-chloropyrid-4-yl]-benzamide) and pumafentrine ((-)-cis-9-ethoxy-6-(4-diisopropylaminocarbonyl-phenyl)-8-methoxy-2-methyl-1,2,3,4,4a,10b-hexahydro-benzo^hello^c]^hello^1,6]naphthyridine-hydrochloride) were synthesized by the Department of Chemistry at the Nycomed GmbH, Konstanz, Germany, a member of the Takeda Group. The compounds were suspended in 4% methylhydroxylpropylcellulose with PEG400 (methocel; Dow Chemicals, Midland, USA) diluted in distilled water to final concentrations for oral dosing of 1 mg/kg/d or 5 mg/kg/d body weight for roflumilast and 1.5 mg/kg/d or 5 mg/kg/d for pumafentrine.

### Induction of Colitis and Treatment

Mice were fed 3.5% DSS (molecular weight 30–40 kDa; ICN, Eschwege, Germany) dissolved in sterile, distilled water ad libitum throughout the experimental period (days 1–11). The DSS concentration was determined in previous dose finding studies for Balb/c mice [24]. The animals within each group showed a comparable severity of colitis and the amount of consumed drinking water per animal was similar in all experimental groups – independently of treatment. Roflumilast, pumafentrine, and 4% methocel (vehicle) were freshly prepared and administered orally (by gavage) once daily in a volume of 200 µl in parallel with DSS-feeding. Control mice had free access to tap water. They once daily received oral roflumilast, pumafentrine, or 4% methocel for a total of 11 days. The pharmacological doses were selected based on previous *in vivo* experiments [22], [25].

### Determination of Clinical Score, Colon Length and Histologic Score

Body weights, as well as stool consistency and occult blood or the presence of gross blood per rectum were determined daily. Two investigators blinded to the protocol independently assessed the clinical score as previously described [9], [10], [24]. Briefly, weight loss of 1–5%, 5–10%, 10–20%, and >20% was scored as 1, 2, 3, and 4, respectively. For stool consistency, 0 was scored for well-formed pellets, 2 for pasty and semiformed stools, which did not stick to the anus, and 4 for liquid stools that remained adhesive to the anus. Bleeding was scored 0 for no blood in hemoccult, 2 for positive hemoccult, and 4 for gross bleeding from the rectum. Weight, stool consistency, and bleeding sub-scores were added and divided by 3, resulting in a total clinical score ranging from 0 (healthy) to 4 (maximal activity of colitis). Post mortem the entire colon (cecum to anus) was removed from the caecum to the anus and the colon length was measured as an indirect marker of inflammation. Rings of the transverse part of the colon were fixed in 10% formalin and embedded in paraffin for histologic analysis. Sections (4 µm) were stained with hematoxylin & eosin. Histologic scoring was performed based on the extent of infiltration of inflammatory cells: 0 for very few inflammatory cells in the lamina propria; 1 for increased numbers of inflammatory cells, including neutrophils in the lamina propria; 2 for confluence of inflammatory cells, extending into the submucosa; and 3 for extension through deeper structures of the bowel wall. In addition tissue damage was assessed and scored from 0 (no tissue damage) to 3 (most severe tissue damage). The two sub-scores for inflammation and tissue damage were added and the combined histologic score ranged from 0 (no changes) to 6 (extensive cell infiltration and tissue damage).

### Colon TNFα Extraction

Strips (about 4 cm) of colon from DSS-exposed and from non-DSS exposed mice with and without roflumilast or pumafentrine treatment were weighed, vigorously vortexed for 1 min in 100 µl of PBS (Roche, Ingelheim, Germany) and centrifuged at 10.000x at 4°C for 15 min. The supernatants (eluates) were stored at –70°C until further use.

### Cytokine Detection

TNFα in the colonic supernatants and IFNγ in the medium of cell cultures were quantified with a commercial enzyme-linked immunosorbent assay kit (Endogen, Woburn, USA) according to the manufacturer’s instructions. The lower limit of detection of both assays was 50 pg/ml.

### Cell Culture and Flow Cytometry

Spleens were aseptically removed from pumafentrine-treated mice at day 11 and weighed. Cell suspensions were prepared according to standard procedures [26]. Cells were washed twice in RPMI-1640, resuspended in medium containing 10% fetal calf serum, and cultured at a concentration of 2.5×10^6^ cells/ml in 48-well plates. Cultures were incubated for 20 h in the presence or absence of phorbol myristate acetate (PMA, 25 ng/ml, Sigma, Munich, Germany) plus ionomycin (500 ng/ml, Sigma) at 37°C in a humidified atmosphere with 5% CO_2_. At the end of the incubation period half of the culture medium and cells were frozen at –70°C until cytokine measurement. The remaining cultures were used for flow cytometry analysis (FACS Calibur; Becton Dickinson, Heidelberg, Germany). Flow cytometry followed routine procedures using 5×10^6^ splenocytes/sample. To determine the expression of CD69, CD45R, CD3 and Mac, cells were labeled with either fluorescein isothiocyanate- or phycoerythrine-labeled specific antibodies (Becton Dickinson).

### Statistical Analysis

Biostatistical analysis of the data was performed on the basis of means. Monotone dose dependency for the inhibition of the clinical score was evaluated by the nonparametric Jonckheere Terpstra test using the individual data at each investigated time point. This test is similar to the Kruskal-Wallis test in that the null hypothesis is that several independent samples are from the same population. However with the Kruskal-Wallis test there is no a priori ordering of the populations from which the samples are drawn. The Jonckheere Terpstra test has more statistical power than the Kruskal-Wallis test when there is a-priori ordering and therefore has been used for this study. All other parameters (colon length, histologic score, cytokine concentration in colonic tissue, cytokine production, or CD69 expression by spleen cells) were tested also by the Jonckheere Terpstra test at a given time point after sacrifice with a α level of 0.05. Data are presented as mean +/− SEM.

## Results

### Roflumilast Mitigates Experimental Colitis in Mice

#### Clinical score and colon length

**Figure 1:**
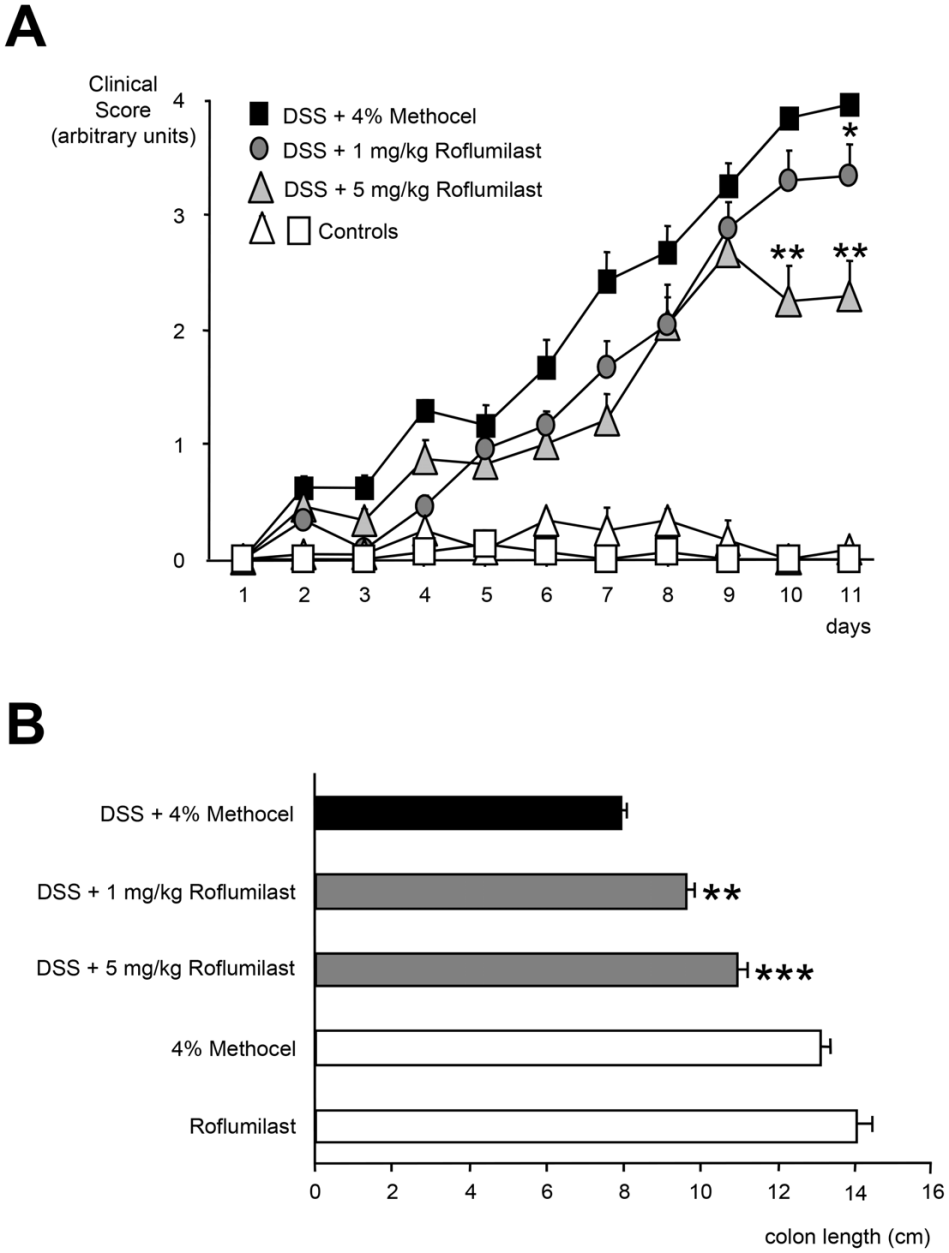
Effect of roflumilast on clinical score and colon length. A. Mitigation of DSS-induced colitis by roflumilast. Mice were exposed to 3.5% DSS in drinking water for 11 days. Either 1 mg/kg/d roflumilast, 5 mg/kg/d roflumilast or 4% methocel were administered orally once daily for 11 days (n = 8). Non-DSS-treated mice received 5 mg/kg/d roflumilast (n = 8) or 4% methocel (n = 5). The degree of colitis was quantified by the clinical score assessing weight loss, stool consistency and rectal bleeding (range from 0 =  healthy to 4 =  maximal disease activity). Scores are depicted as mean ± SEM; *p<0.05, **p<0.01 versus DSS+methocel. **B. Effect of roflumilast on colon length shortening in DSS-induced colitis.** Mice were exposed to 3.5% DSS in drinking water for an 11 day period. Roflumilast treatment (either 1 mg/kg/d or 5 mg/kg/d orally, once daily for eleven days) or 4% methocel were started on the same day as DSS administration (n = 8). Non-DSS mice received 5 mg/kg/d roflumilast (n = 8) or 4% methocel (n = 5). Values are depicted as mean ± SEM. **p<0.01, ***p<0.001 versus DSS+methocel.

Mice exposed to 3.5% DSS developed signs of colitis as expressed by a clinical score higher than 0.5 starting on day 2 **(** **Figure 1A** **)**. Treatment with 5 mg/kg/d roflumilast halted the progression of colitis as expressed by a lower clinical score versus the roflumilast 1 mg/kg/d group and the control group, respectively, starting from day 10. The difference persisted illustrating no further progression of colitis at the point of the last measure on day 11 for the roflumilast 5 mg/kg/d group (p<0.01). On day 11, the difference was also significant for the roflumilast 1 mg/kg/d group (p<0.05; n = 8 per group; **Figure 1A**). In the animals exposed to DSS, each of the three clinical parameters was independently improved by administration of 5 mg/kg/d roflumilast (data not shown). Mice not exposed to DSS and treated with either 5 mg/kg/d roflumilast or 4% methocel did not show any signs of colitis (n = 5).

Colon length is an indirect and reproducible morphologic parameter for the severity of colonic inflammation (Siegmund et al. 2001; Loher et al. 2003). Roflumilast partially and dose-dependently reversed the DSS-induced about 40% reduction in colon length by 31% (p<0.01) and 58% p<0.001) at 1 and 5 mg/kg/d dose levels, respectively (n = 8 each group). The control groups not exposed to DSS and treated with 5 mg/kg/d roflumilast or 4% methocel showed the greatest colon length (n = 5), respectively (**Figure 1B**).

#### Histologic score

**Figure 2:**
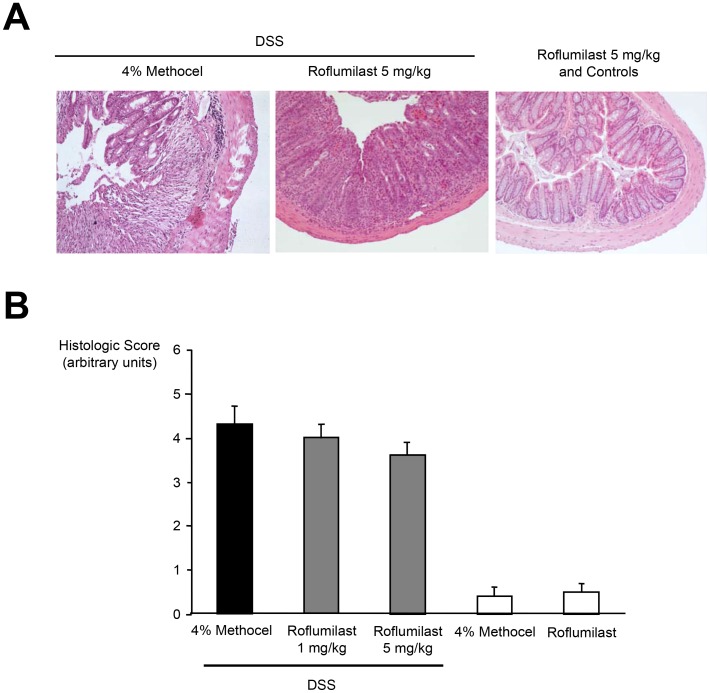
Effect on histologic signs of colonic inflammation by roflumilast. Mice were exposed to 3.5% DSS in drinking water for 11 days and were treated with roflumilast (either 1 or 5 mg/kg/d orally once daily for 11 days, n = 8) or 4% methocel (n = 5). Non-DSS mice received 5 mg/kg/d roflumilast (n = 8) or 4% methocel (n = 5). At day 11 mice were euthanized, colon rings were stained and the histologic score (degree of inflammation and tissue damage: 0 = no changes to 6 = extensive cell infiltration and tissue damage) was determined in a blinded fashion as described in the *Materials and Methods*. **A.** Representative cross sections of the transversing colon. Magnification of the images is 200-fold. **B.** Scores are depicted as means ± SEM.

Histology of rings of the transverse part of the colon in DSS-exposed mice revealed multiple erosive lesions and inflammatory cell infiltration composed of mainly macrophages with fewer lymphocytes and occasional eosinophils and neutrophils **(** **Figure 2A** **)**. After 11 days of continuous DSS administration, treatment with roflumilast showed a tendency to decrease the histologic score as compared to the DSS control group **(** **Figure 2B** **)**. In the control groups not exposed to DSS, no histologic signs of inflammation were detected.

#### TNFα concentration in colonic tissue

**Figure 3:**
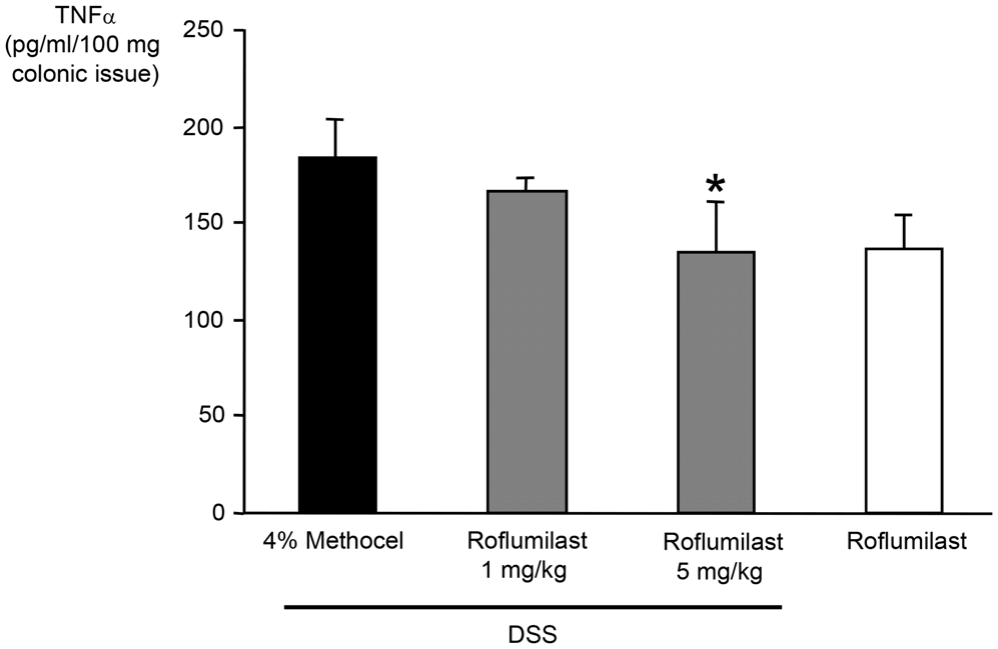
Reduction of colonic mucosa TNFα content by roflumilast. Mice were exposed to 3.5% DSS in drinking water for eleven days and were treated with roflumilast (either 1 or 5 mg/kg/d orally once daily for 11 days, n = 8) or 4% methocel (n = 5). At day 11 the colon was removed, weighed, vortexed in PBS and centrifuged. TNFα was quantified in the eluate by ELISA. Values represent mean ± SEM; *p<0.05.

TNFα concentration in the colonic tissue was reduced in roflumilast (5 mg/kg/d)-treated DSS-mice on day 11 compared to the 4% methocel-treated DSS group (**Figure 3**; n = 5). Colons of control mice not being exposed to DSS but receiving roflumilast showed mean TNFα concentrations comparable to the DSS/roflumilast group (**Figure 3** **)**. Preliminary experiments showed that the colonic TNFα concentration in control animals receiving regular water was not different if PDE4 was inhibited or not (data not shown).

### Pumafentrine Alleviates Experimental Colitis in Mice

The therapeutic potential of the PDE4 inhibitor roflumilast in DSS-induced colitis led us to evaluate the effect of a dual selective PDE3/PDE4-inhibition with pumafentrine in this model.

#### Clinical score and colon length

**Figure 4:**
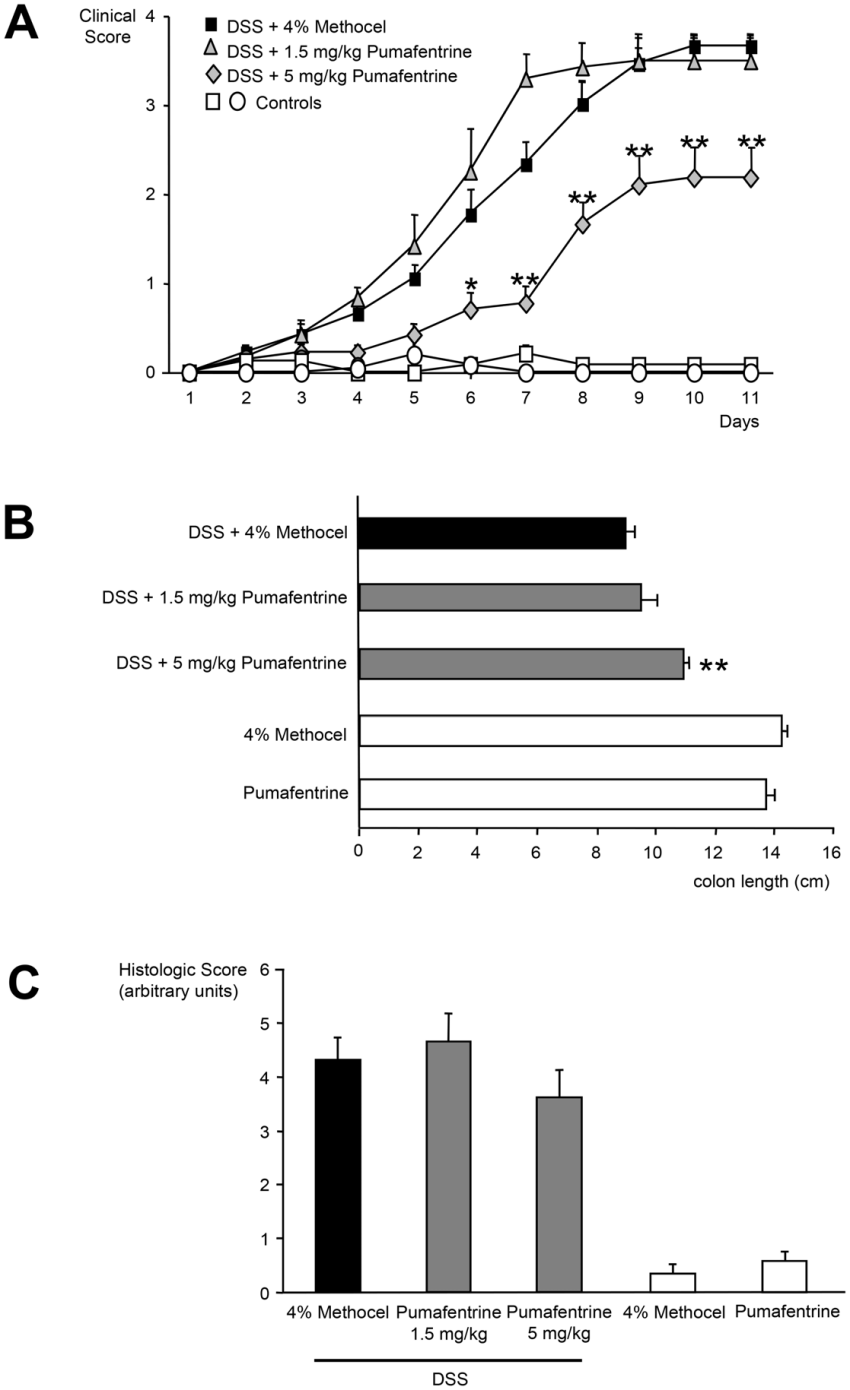
Effect of pumafentrine on clinical score, colon length and histologic score. A. Efficacy of pumafentrine in DSS-induced colitis. Mice were exposed to 3.5% DSS in drinking water for eleven days. Either 1.5 mg/kg/d pumafentrine (n = 8), 5 mg/kg/d pumafentrine (n = 16) or 4% methocel (n = 16) were administered orally once daily for eleven days. Non-DSS mice received either 20 mg/kg/d pumafentrine or 4% methocel (n = 8). The degree of colitis was quantified by the clinical score assessing weight loss, stool consistency and rectal bleeding (range from 0 =  healthy to 4 =  maximal disease activity). The scores are depicted as mean ± SEM; *p<0.05, **p<0.01 versus DSS+methocel. **B. Reduction of colon length shortening in DSS-induced colitis by pumafentrine.** Mice were exposed to 3.5% DSS in drinking water for an eleven day period. Pumafentrine treatment (either 1.5 mg/kg/d (n = 8) or 5 mg/kg/d (n = 16) orally, once daily for 11 days) or 4% methocel (n = 12) were started the same day as DSS administration. Non-DSS mice received 20 mg/kg/d pumafentrine or 4% methocel (n = 8), respectively. Values are depicted as mean ± SEM. **p<0.01 versus DSS+methocel. **C. Effect on histologic signs of colonic inflammation by pumafentrine.** Mice were exposed to 3.5% DSS in drinking water for eleven days and were treated with pumafentrine (either 1.5 mg/kg/d (n = 8) or 5 mg/kg/d (n = 16) orally once daily for 11 days) or 4% methocel (n = 12). Non-DSS mice received 20 mg/kg/d pumafentrine or 4% methocel (n = 8). At day 11 mice were euthanized, colon rings were stained and the histologic score (degree of inflammation: 0 =  no changes to 6 =  extensive cell infiltration and tissue damage) was determined in a blinded fashion as described in detail in the *Material and Methods*. Scores are depicted as means ± SEM.

Mice exposed to 3.5% DSS developed signs of colitis as expressed by a clinical score of greater than 0.5 from day 4 onward **(** **Figure 4A** **)**. Oral administration of pumafentrine 5 mg/kg/d retarded the onset of colitis to day 6. No dose dependency of the treatment was noted starting day 8. Treatment with pumafentrine 5 mg/kg/d resulted in a lower clinical score starting at day 6 of the experiment and lasting until the end of the experiment on day 11 (n = 16; **Figure 4A**). Administration of 1.5 mg/kg/d pumafentrine did not delay the onset of clinical signs of colitis and also did not influence the clinical score until day 11 when compared to the untreated DSS animals. The non-DSS control animals, which received regular drinking water and pumafentrine 5 mg/kg/d, 20 mg/kg/d, or 4% methocel (n = 8) did not develop signs of colitis during the experimental course (all clinical scores <0.5 on days 1 to 11).

Colon length, a surrogate for colitis severity, was significantly longer in the 5 mg/kg/d pumafentrine group as compared to the control group. This represents a 36% reversal by pumafentrine of a 37% shortening in the DSS group. DSS-exposed animals treated with 1.5 mg/kg/d pumafentrine did not show any significant difference compared to animals treated with DSS only. In the control groups, the highest colon length was observed (**Figure 4B**).

#### Histologic score

Histologic analysis of the rings of the transverse part of the colon in DSS-fed mice revealed multiple erosive lesions and inflammatory cell infiltrations. After 11 days of continuous DSS administration, treatment with 5 mg/kg/d pumafentrine showed a tendency to decrease the histologic score compared to the 4% methocel-treated group, an effect that did not reach statistical significance **(** **Figure 4C** **)**. In non-DSS control animals, no signs of inflammation could be detected. Animals treated with pumafentrine 20 mg/kg/d as well as those receiving methocel had similarly low scores.

#### TNFα concentration in colonic tissue

**Figure 5:**
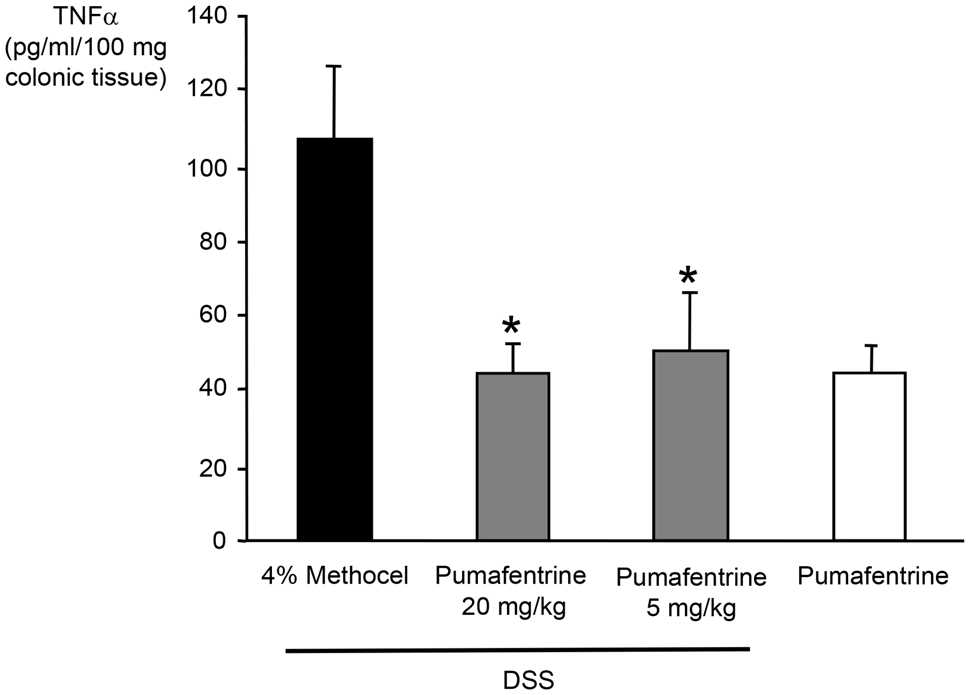
Effect of pumafentrine on colonic mucosa TNFα concentration. Mice were exposed to 3.5% DSS in drinking water for 11 days and were treated with pumafentrine (either 1.5 mg/kg/d (n = 8) or 5 mg/kg/d (n = 16) orally once daily for 11 days) or 4% methocel (n = 12). Non-DSS mice received 20 mg/kg/d pumafentrine or 4% methocel (n = 8). At day 11, the end of experiment, the colon was removed, weighed, vortexed in PBS and centrifuged. TNFα was quantified in the eluate by ELISA. Values represent mean ± SEM; *p<0.05 versus DSS+methocel.

For measurement of colonic tissue TNFα concentrations the colons of mice treated as described above were obtained at day 11. The TNFα concentration in the colonic tissue was reduced in pumafentrine-treated DSS-mice compared to the methocel group at day 11 (**Figure 5**). Colons displaying no signs of inflammation in the pumafentrine control groups showed low mean TNFα concentrations **(** **Figure 5** **)**.

#### IFNγ production by cultured splenocytes

**Figure 6:**
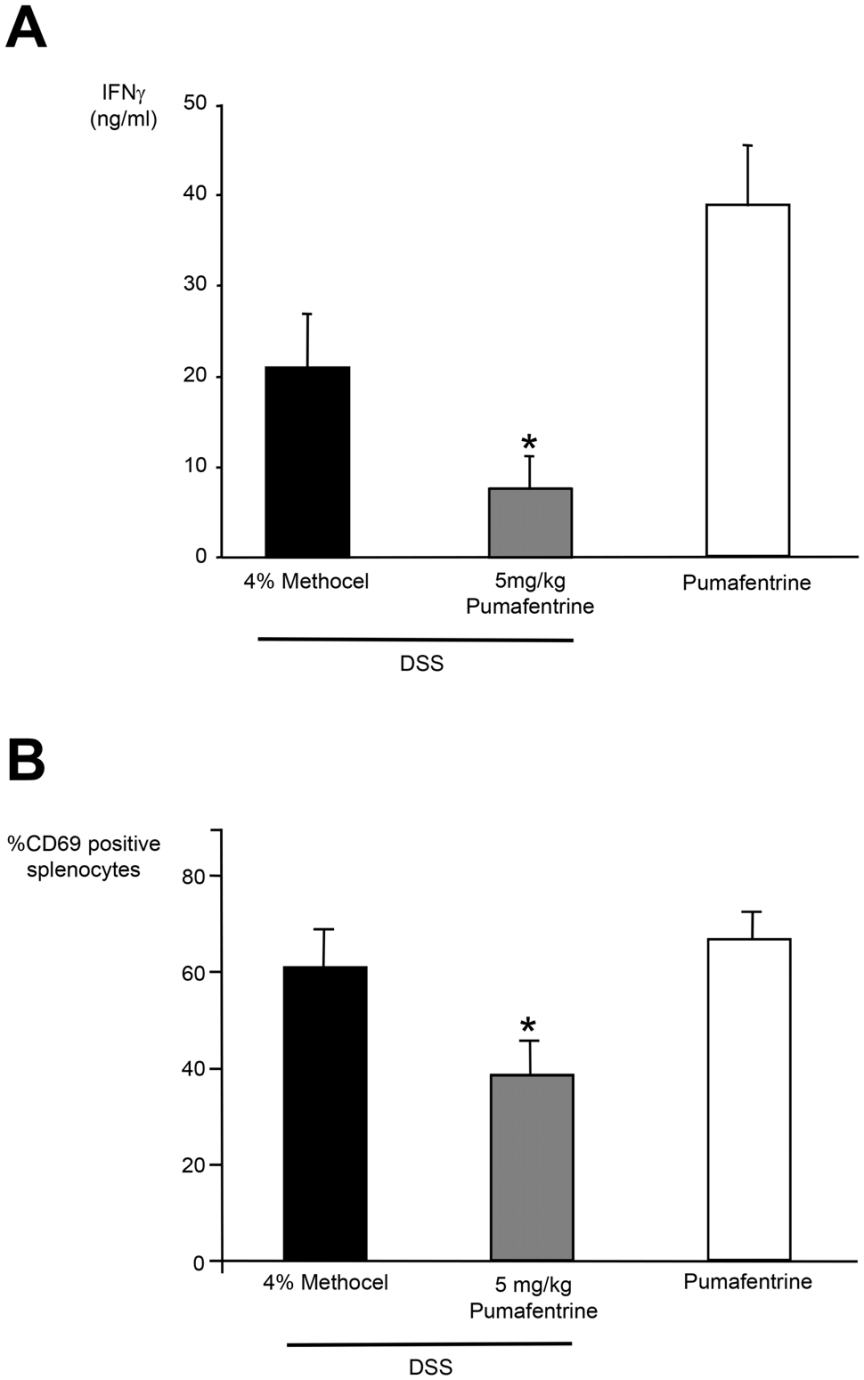
Systemic effects of pumafentrine. A Effect of *in vivo* administered pumafentrine on PMA/ionomycine-induced IFNγ synthesis by splenocytes from DSS-exposed mice. Mice were exposed to 3.5% DSS in drinking water for 11 days and were treated with pumafentrine 5 mg/kg/d (n = 8) orally once daily for 11 days) or 4% methocel (n = 8). Non-DSS mice received 20 mg/kg/d pumafentrine (n = 8). After day 11 spleens were removed aseptically and splenocytes were isolated. After a 20 h incubation period with PMA 25 ng/ml and ionomycine 500 ng/ml, the experiment was stopped by three freeze-thaw cycles and total IFNγ was measured by ELISA. Results are depicted as mean ± SEM. *p<0.05 versus DSS+methocel. **B. Effect of** ***in vivo*** **administered pumafentrine on PMA/ionomycine-induced CD69 expression by splenocytes from DSS-exposed mice.** Mice were exposed to 3.5% DSS in drinking water for 11 days and were treated with pumafentrine 5 mg/kg/d (n = 8) orally once daily for 11 days) or 4% methocel (n = 8). Non-DSS mice received 20 mg/kg/d pumafentrine (n = 8). After day 11 spleens were removed aseptically and splenocytes were isolated and stimulated with PMA 25 ng/ml and ionomycine 500 ng/ml. After 20 h incubation period the CD69-expression was determined by flow cytometry. Bars represent the mean ± SEM of CD69 positive splenocytes. *p<0.05 versus DSS+methocel.

As dual selective PDE3/PDE4 inhibitors have never been tested in a preventive colitis model we evaluated the effects of *in vivo*-administered pumafentrine on IFN-γ production *in vitro*. Splenocytes at the end of day 11 were cultured for 20 h with PMA (25 ng/ml) plus ionomycine (500 ng/ml) or without stimulus. PMA plus ionomycine stimulated splenocytes from the methocel-treated DSS group showed a higher IFNγ production as compared to the pumafentrine-treated animals (**Figure 6A**). Controls not exposed to DSS showed the highest IFNγ secretion (20 mg/kg/d pumafentrine group). In preliminary experiments no significant difference in IFNγ production between control animals receiving pumafentrine or water was detected. The unstimulated splenocytes did not produce detectable levels of IFNγ (data not shown).

#### CD69 expression by cultured splenocytes

Splenocytes were also examined by flow cytometry (**Figure 6B**) for expression of CD69, an activation marker for a wide variety of immune cells. The investigated splenocyte cell population consisted of 50% lymphocytes (CD45R^+^), 32% T-cells (CD3^+^), 7% macrophages (Mac-1^+^) and 11% cells of unknown specificity (data not shown). Within the DSS exposed group and after stimulation with PMA plus ionomycin for 20 h the splenocytes from animals receiving methocel showed increased expression of CD69 compared with cells derived from animals receiving pumafentrine. In animals not exposed to DSS and treated with 20 mg/kg/d pumafentrine values of CD69 expression were comparable to the DSS methocel group **(** **Figure 6B** **)**. Less than 6% of unstimulated splenocytes were positive for CD69 independent of the various treatments. There was no significant difference in CD69 expression on splenocytes in the control groups between the pumafentrine and 4% methocel control animals.

The relative improvement for the endpoints used after treatment with both substances is summarized in **Table 1**.

**Table 1:** Relative improvement of experimental colitis with roflumilast 5 mg/kg/d and pumafentrine 5 mg/kg/d.

	DSS-roflumilast (5 mg/kg/d) versusDSS-methocel (% improvement)	DSS-pumafentrine (5 mg/kg/d) versusDSS-methocel (% improvement)
**Clinical score (day 7)**	50±3	65±5 **
**Clinical score (day 11)**	43±7 **	41±7 **
**Colon length (day 11)**	38±1 ***	15±3 **
**Histologic score (day 11)**	16±4	14±3
**Colonic TNF**α**-levels (day 11)**	27±6 *	54±6 *

Data is depicted as mean ± standard error * p<0.05,

** p<0.01,

*** p<0.001, DSS: Dextrane sodium sulfate; TNFα: tumor necrosis factor-α.

Data are depicted as % improvement of the dextrane sulfate sodium (DSS) treatment groups versus DSS methocel groups.

## Discussion

In the present study, we demonstrated the *in vivo* effects of once daily oral administration of the PDE4 inhibitor roflumilast and the PDE3/PDE4 inhibitor pumafentrine in the prevention of DSS-induced colitis. Treatment with roflumilast dose-dependently ameliorated the clinical score, led to a reduced shortening of the colon length and decreased concentration of TNFα in colonic tissue. However, this improvement was not associated with a lower histologic score. Pumafentrine at a dose of 5 mg/kg/d reduced the clinical score and partially reversed colon shortening and TNFα synthesis in colonic tissue. There was a tendency to improve the histopathological colonic changes. As a systemic effect of *in vivo* treatment with pumafentrine, isolated splenocytes *ex vivo* synthesized less IFNγ and expressed lower amounts of CD69 on the cell surface after stimulation with PMA and ionomycine than splenocytes derived from control animals.

In the testing of novel therapeutics the DSS-induced colitis model, used in the current study, has a number of advantages, including its simplicity and the high degree of uniformity and reproducibility of the colonic lesions [23]. The cytokine profile and histopathology of murine DSS-colitis has similarities with both forms of IBD, namely elevation of pro-inflammatory cytokines, such as TNFα and IFNγ, transmural inflammation, and aphthous erosions (like CD) as well as increased levels of IL-4 and crypt abscesses (like UC). The DSS model of murine colitis has been proven useful for preclinical testing of new compounds for the therapy of human IBD [23], [27], [28], [29].

Among the PDE isoforms PDE3 and PDE4 were identified as the predominant cAMP-hydrolyzing PDE in human inflammatory cells. Elevation of intracellular cAMP by inhibition of PDE4 is associated with a broad anti-inflammatory activity *in vitro*, including suppression of TNFα and IFNγ, and induction of IL-10 [7], [11], [15]. A combined inhibition of PDE3 and PDE4 may result in overadditive effects on anti-inflammatory cellular functions [15].

Early PDE4 inhibitors have successfully been tested in animal models of experimental arthritis, autoimmune encephalomyelitis, and respiratory inflammatory diseases, where PDE4 inhibitors resulted in reduced inflammatory cell infiltrates and expression of pro-inflammatory cytokines [13], [30], [31]. In humans, however, the clinical use of early PDE4 inhibitors was limited by their tolerability profile. Roflumilast showed anti-inflammatory activity in animal models and in humans, was more potent than the early PDE4 inhibitors rolipram and cilomilast [16], [25], but was well tolerated [21].

By combining PDE3 and PDE4 inhibitors *in vivo* the benefits of over-additive effects may apply as described *in vitro*. Dual selective inhibitors of PDE3 and PDE4 have been studied in animal models of asthma (benafentrine and zardaverine) [32] or experimental pulmonary hypertension (pumafentrine) [22].

Recent data from the literature suggest that inhibition of PDE4 might provide a novel approach in the therapy of IBD in humans. The PDE4 inhibitors rolipram, mesopram and tetomilast improved DSS-colitis in a preventive, therapeutic and chronic setting [9],[10],[33],[34]. Rolipram had a stronger anti-inflammatory activity compared to pentoxyphylline in the acute DSS-colitis model [35]. In humans the oral once daily administration of tetomilast (OPC-6535) has been tested for mild to moderately active UC in a randomized controlled trial. While a post hoc analysis suggested efficacy of tetomilast in UC patients with high disease activity, the primary end point (improvement of clinical disease activity) did not reach statistical significance [36].

In our study, we focused the investigations of the selective PDE4 inhibitor roflumilast and the dual-selective PDE3/4 inhibitor pumafentrine on two endpoints. First, local effects at the sites of inflammation were examined. There, the clinical activity was determined with a semiquantitative scoring system that has been described as a reliable marker of pathologic changes during the disease course [9], [10], [24], [37]. Roflumilast dose-dependently alleviated the clinical course of colitis. Pumafentrine improved the clinical score at the dose of 5 mg/kg/d. Comparable results were seen in the shortening of the colon as a morphometric surrogate for the degree of inflammation. TNFα is a key cytokine in human IBD. The local TNFα expression was measured *ex vivo* as described previously [10], [24]. Roflumilast and pumafentrine both significantly reduced the synthesis of TNFα in the colon. However this suppression was stronger in the pumafentrine (54%) versus roflumilast (27%) treated animals. These observations may further support that TNFα is only one example for cytokines being involved in this experimental model of colitis, amongst other inflammatory mediators possibly modulated by the PDE inhibitor (e.g. IL-2, IL-10 or IFNγ). Further studies should address this hypothesis, by testing the involvement of additional cytokines in this model. Surprisingly, no significant change on the histologic score was observed with both substances. This was not in concordance with previous reports, which showed a close relation between crypt lesions and clinical activity [37]. This could be explained by the high degree of inflammation in all DSS-exposed animals in this experimental series. An alternate explanation could be an insufficient efficacy of the tested substances in experimental colitis. While surrogate parameters of inflammation were reduced, this improvement was not reflected by the histologic score, suggesting that additional factors, aside a reduction in TNFα levels could be responsible for the observed tissue damage.

As PDE3/PDE4 inhibitors have not previously been tested in a preventive colitis model we examined the systemic effect of pumafentrine by investigation of splenocyte phenotype and function. As endpoints of these experiments IFNγ, a cytokine released upon natural killer cell and T-cell activation, and CD69, one of the earliest cell surface antigens induced on activated T cells, thymocytes, B cells, natural killer cells, and neutrophils were chosen [38]. Both IFNγ synthesis and expression of CD69 were markedly suppressed in the pumafentrine group compared to the group exposed to DSS only. This finding was consistent with the reduced clinical score, the shortening of the colon length, and TNFα expression in the colon. No inhibitory effect on IFNγ synthesis or CD69 expression by pumafentrine treatment was detected in mice not exposed to DSS (data not shown).

These data indicate that elevation of intracellular cAMP influences the regulation of IFNγ and CD69. Nonetheless, these results cannot be explained by a direct influence of pumafentrine as *ex vivo* all pharmacologic substances were washed out during the isolation process. It is known that activation of adenylate cyclase by autocrine mediators such as prostaglandin E2 (PGE2) or prostacyclin may have a synergistic effect with PDE inhibition to augment cAMP and reduce inflammatory cellular effects [39]. In the inflamed mucosa of IBD patients, PGE2 and prostacyclin concentrations are elevated. Therefore, oral administration of specific PDE inhibitors might lead to the strongest effect locally in the gut. IFNγ synthesis was higher in stimulated splenocytes of mice not exposed to DSS as compared to DSS-exposed mice. This might be due to a desensitization of splenocytes during the systemic inflammatory response, as described for LPS-induced desensitization in murine monocytes [40]. In addition, due to the absence of inflammatory mediators such as PGE_2_ and prostacyclin, pumafentrine might not have been able to exert its synergistic effects leading to a preservation of the IFNγ producing cell pool. A similar phenomenon was seen by treatment with the adenosine kinase inhibitor GP515 (also an cAMP-elevating agent, [24]) and the PDE4 inhibitor mesopram (data not shown). The lack of efficacy observed for the 1.5 mg/kg/d pumafentrine group was probably due to the low dose.

### Conclusions

This study demonstrated, that the PDE4 inhibitor roflumilast and the PDE3/PDE4 inhibitor pumafentrine dose-dependently ameliorated the clinical score and colonic TNFα production in murine DSS-induced colitis in a preventive setting, while not affecting the histopathologic colonic findings. Roflumilast has been investigated in largescale clinical studies in patients with COPD, showing anti-inflammatory activity leading to a reduction of COPD exacerbations and improvement of the FEV1 with acceptable tolerability. PDE4 or PDE3/4 inhibitors might represent potential tools for the anti-inflammatory therapy of IBD. Future studies should address additional mechanisms of action of these PDE inhibitors, focussing on their anti-inflammatory or cytoprotective activity in intestinal inflammation.
